# Antimicrobial, antioxidant and anti-tyrosinase properties of extracts of the Mediterranean parasitic plant *Cytinus hypocistis*

**DOI:** 10.1186/s13104-015-1546-5

**Published:** 2015-10-13

**Authors:** Paolo Zucca, Manuela Pintus, Giorgia Manzo, Mariella Nieddu, Daniela Steri, Andrea C. Rinaldi

**Affiliations:** Department of Biomedical Sciences, University of Cagliari, Cittadella Universitaria, Complesso Universitario, SP Monserrato-Sestu Km 0.700, 09042 Monserrato, CA Italy; Consorzio UNO, Oristano, Italy

**Keywords:** *Cytinus*, *Cynomorium*, Plant extracts, Parasitic plants, Antimicrobial, Antioxidant, Anti-tyrosinase

## Abstract

**Background:**

*Cytinus* is an endophytic parasitic plant occurring in South Africa, Madagascar, and in the Mediterranean region. We have extracted the inflorescences (the only visible part of the plant, emerging from the host roots at the time of blossom) of *Cytinus hypocistis* collected in Sardinia, Italy, and explored the antimicrobial, antioxidant, anti-tyrosinase, and cytotoxic activities of the extracts.

**Methods:**

Extracts from *C. hypocistis* were prepared using increasing polarity solvents: cyclohexane, ethanol, and water. Phenolic composition were determined through spectrophotometric assays, and antioxidant activity with both electron-transfer and hydrogen-atom assays. Nine different bacterial strains, including clinical isolate methicillin-resistant *Staphylococcus aureus*, were used in agar diffusion method. Cytotoxicity was tested using against the B16F10 melanoma cell line.

**Results:**

While cyclohexane extracts where biologically inactive, ethanolic and aqueous extracts displayed an intriguing activity against several Gram-positive bacterial strains, including methicillin-resistant *S. aureus*, and against the Gram-negative *Acinetobacter baumanii*. Compared to the conventional antibiotics like cloxacillin, ampicillin, and oxytetracycline, *C.hypocistis* extracts were less active in absolute terms, but displayed a wider spectrum (notably, cloxacillin and ampicillin were inactive against methicillin-resistant *S. aureus*). The ethanolic extract of *C. hypocistis* was found to be particularly rich in polyphenols, in most part hydrolysable tannins. The antioxidant activity of extracts, tested with several methodologies, resulted to be particularly high in the case of ethanolic extracts, in accordance with the composition in phenolics. In detail, ethanol extracts presented about a twofold higher activity than the water sample when tested through the oxygen radical absorbance capacity-pyrogallol red (ORAC-PYR) assay. Cytotoxicity analysis against the B16F10 melanoma cell line showed that both extracts have not significant cytotoxic effect, even at the highest dose (1000 µg/mL). Tests showed that ethanolic extracts also had the greatest tyrosinase inhibition activity, indicating that *C. hypocistis*-derived substances could find application in food formulations as anti-browning agents.

**Conclusions:**

Overall, these results point to the need of further studies on *C. hypocistis* extracts, aimed at isolating and fully characterizing its biologically active compounds.

## Background

Plants host a trove of active substances, and are the basis of both ancient folk remedies and modern pharmacopeia [1, 2]. Even among the otherwise well studied European flora, the chemical/biochemical grounds of many traditional healthcare practices remains obscure, and many have called for a renaissance of ethnobiological studies—including in this term not only local traditional knowledge regarding medicine sources, but also the environment and wild food—in this part of the Old World [2, 3].

**Fig. 1:**
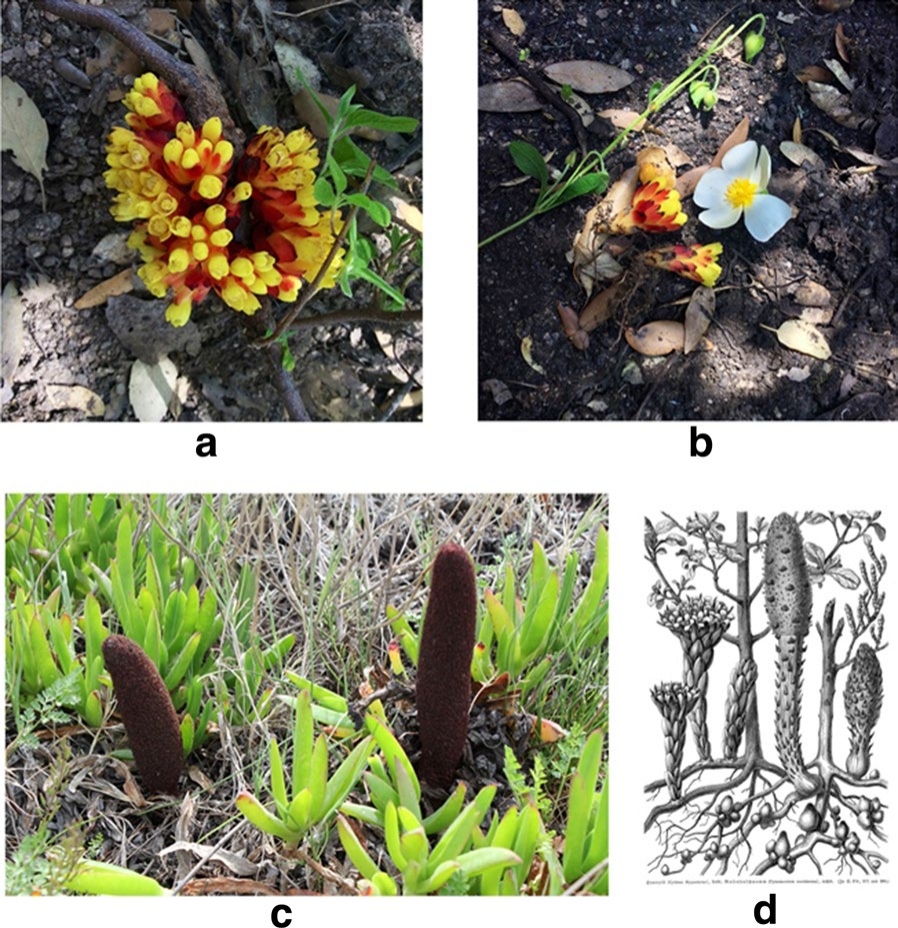
**a** Growth habit of *Cytinus hypocistis* growing on *Cistus salvifolius*, showing a clump of multiple inflorescences arising from a single parasite; **b** *C. hypocistis* inflorescences, side view; **c** *Cynomorium coccineum*, the Maltese mushroom, growing in a coastal area of south-western Sardinia, Italy; **d** a classic portrait of *C. hypocistis* and *C. coccineum*, depicted in the same plate (from [42])

*Cytinus* is a genus composed of 8 recognized species (plus several others under study) of nonchlorophyllic plants [4], that parasitize roots of *Cistus* and *Halimium*, two genera of shrub plants in the family Cistaceae. Members of *Cytinus* are rootless, stemless and leafless; flowers are the only visible part, and just during the reproductive period, when they emerge from host tissues. The genus belongs to family Cytinaceae, together with *Bdallophyton* and *Bdallophytum*, and occurs in the Mediterranean region, in South Africa and in Madagascar. The reproductive and trophic biology of *Cytinus* is intriguing. Flowers of the Mediterranean *C. hypocistis* (L.) L. (Fig. 1a, b) are visited by ants [5], while in South Africa *C.sanguineus* is pollinated by sunbirds [6] and mammalian pollination has been demonstrated for *C. visseri* [7]. In this latter case, rodents and elephant shrews seem to be attracted by two aliphatic ketones that dominate the scent of parasite’s flowers [7]. A novel trophic interaction involving *C. hypocistis*, Cistaceae host species, and mycorrhizal fungi, with mycorrhizae being associated with both the hosts and the parasites, was recently described [8]. If confirmed [9, 10], this would represent a very rare, if not unique, case of a tripartite association involving an endophytic parasitic plant, its host, and mycorrhizae in natural conditions [11].

In traditional medicine, a decoction of *C. hypocistis* has been used in the treatment of dysentery and for its astringent qualities; other reported uses include the treatment of tumors of the throat and of eye inflammations, and as a emmenagogue [12, 13]. The young plant can be cooked as an asparagus substitute, flowers sucked as sweets, and the species is quoted as famine food in Portugal [14–16]. In Turkey, the plant was used to produce a glue [17]. In Sardinia, a recent ethnobotanical survey conducted in a territory situated in the south-central part of the island found that the *Cytinus* juice has been used in popular medicine as an astringent, tonic, and haemostatic substance [18].

Despite this wealth of traditional uses, the chemical composition of *Cytinus* is largely unknown, and active substances not identified. To help bridging this gap, and to explore alternative potential medicinal, cosmetic and/or nutraceutical uses of these plants, we here report the antimicrobial, antioxidant, anti-tyrosinase, and cytotoxic activities of extracts of *C. hypocistis* collected in Sardinia, Italy, at the core of the Mediterranean center of diversification of the genus. To the best of our knowledge, this is the first report of a screening of antibacterial activities for *C. hypocistis*, while only antimalarial and antitumoral activities have been reported [19, 20].

## Methods

### Chemicals and instrumentation

All reagents were of the best commercial grade available and used without further purification. Ethanol, cyclohexane and dimethyl sulfoxide were analytical grade solvents obtained from Sigma-Aldrich, Fluka (Milan, Italy). Ultrapure water (18 mΩ) was obtained with a Milli-Q Advantage A10 System apparatus (Millipore, Milan, Italy). Spectrophotometric measurements were carried out with an UltroSpec 2100pro (Amersham Bioscience, Milan, Italy).

### Plant materials

Fresh inflorescences of *Cytinus hypocistis* were collected under host plant, *Cistus salvifolius*, in the vast forested area that extends between Capoterra and Santadi, about 20 km southwest of Cagliari (Sardinia, Italy), on May 2014. Vouchers are kept in the collection of the Department of Biomedical Sciences at the University of Cagliari, with the accession number ACR-14/5/2014. Specimens were transferred to the laboratory within 1 h from harvest and freeze dried immediately. Reference material was deposited in the collection of the Department of Biomedical Sciences, University of Cagliari, Cagliari, Italy.

### Preparation of the extracts

Samples of 1 g of freeze-dried *C. hypocistis* were suspended in 5 mL cyclohexane and left under mechanical agitation for 30 min. The supernatant was collected after centrifugation at 5000*g* for 10 min. The extraction procedure was repeated 4 times. The extracts were collected, lyophilized and stored at 4 °C. The solid powder was then dried by rotary evaporator, and the whole procedure was repeated using as solvent ethanol, and lastly water, to obtain three extracts of different polarity.

### Antimicrobial activity

Antibacterial activity of the extracts was determined against four Gram-negative and five Gram-positive reference bacterial strains, respectively: *Staphylococcus aureus* DSM 1104, methicillin-resistant *Staphylococcus aureus* (MRSA), *Staphylococcus epidermidis* DSM 1798, *Enterococcus faecalis* DSM 2570, *Escherichia coli* DSM 1103, *Enterobacter cloacae* DSM 30054T, *Pseudomonas aeruginosa* DSM 1117, *Acinetobacter baumannii* DSM 30007T, *Klebsiella pneumoniae* DSM 681. A turbidity equivalent to a 0.5 McFarland standard was prepared by direct saline suspension of isolated colonies selected from an 24-h agar plate. The disc diffusion method was used to carry out the antimicrobial activities of extracts according to CLSI procedures [21, 22]. Inoculum suspensions of each strain were swabbed on the top of the MHA plate prepared with 25 mL of Mueller–Hinton agar (MHA), and 0.5 mg of dry extract was added to each disk separately, in addition to the control disk. DMSO was used as negative control. Rifampicin, cloxacillin, ampicillin, and oxytetracycline were used as positive controls.

### Phenolics determination

Soluble phenolics content was determined using a spectrophometric approach: 1 mL of each sample was treated with 2.5 mL Na_2_CO_3_ 2 % *w/v*. After 1 min incubation at 25 °C, 0.25 mL 1 N Folin-Ciocalteu reagent was added. The mixture was incubated at 25 °C in the dark for 45 min and absorbance at 760 nm measured. Gallic acid was used as the standard (linearity range 0.05–0.6 mM), and the results were calculated as gallic acid equivalents (mM GAE). Quantification of total flavonoids was performed using a spectrophotometric method [23, 24]. Aliquots of 0.25 mL of sample were incubated for 5 min at 25 °C in the presence of 1.25 mL H_2_O and 0.075 mL NaNO_2_ (5 % *w/v*). Then, 0.15 mL AlCl_3_ (10 % *w/v*) was added. After 6 min, samples were alkalinized using 0.5 mL of 1 M NaOH and 0.275 mL H_2_O. Absorbance at 510 nm was then measured. Catechin was used as the standard (linearity range 0.1–0.6 mM) and results were expressed as Catechin Equivalent (mM CE), using a standard curve. Total anthocyanin content was determined through spectrophotometric assay, based on differential pH absorbance [25]. HCl/KCl 0.2 M and sodium acetate 1 M buffers were used to record absorbance at pH 1.0 and 4.5, using both 510 and 700 nm wavelengths. Molar extinction coefficient of cyanidin 3-*O*-glucoside (29,300 M^−1^ cm^−1^) was then used to calculate total anthocyanin, according to Eq. 1 (*l* = optical path).

1 $$[total\;anthocyanin] = \frac{{[(A_{510} - A_{700} )_{{{\text{pH}}\;1.0}} - (A_{510} - A_{700} )_{{{\text{pH}}\;4.5}} ]}}{{29,300\;{\text{M}}^{ - 1} \;{\text{cm}}^{ - 1} \cdot l}}$$

### Determination of antioxidant capacity

- 1,1-Diphenyl-2-picrylhydrazyl radical (DPPH) scavenging assay was performed incubating 0.3 mL of sample and 0.7 mL of DPPH solution (25 mg/L in ethanol). Decrease in absorbance at 515 nm was followed for 30 min at 25 °C. The percentage of DPPH decoloration (%_DEC_) was calculated as follows: %_DEC_ = 100 × [(Abs_control_ − Abs_sample_)/Abs_control_]. Trolox was used for the calibration curve (linearity range 5–50 μM), and results are expressed as Trolox Equivalents (TE) and as IC_50_ [23, 26].
- Ferric reducing antioxidant power (FRAP) was determined at 37 °C. Briefly, 2.5 mL of 10 mM 2,4,6-tripyridyl-*s*-triazine (TPTZ) in 40 mM HCl was incubated with 25 mL of 0.1 M sodium acetate buffer (pH 3.6) and 2.5 mL of 20 mM FeCl_3_. After warming to 37 °C, 0.2 mL of this solution were added to 0.77 mL H_2_O and 0.03 mL of sample. After 6 min at 25 °C, reaction mixtures were centrifuged at 8000*g* for 10 min, and absorbance at 593 nm was measured [24, 26]. The results were expressed as both Trolox Equivalents (mM TE) and mmol of Fe(II) per gram of dry material (mmol Fe^II^/g).
- The trolox equivalent antioxidant capacity (TEAC) assay was performed using 2,2′-azinobis(3-ethylbenzothiazoline 6-sulphonate) (ABTS) cationic radical, obtained by reaction between 7 mmol aqueous ABTS and 2.45 mmol aqueous K_2_S_2_O_8_ at 25 °C for 16 h. Dilution with sodium phosphate buffer 75 mM (pH 7.4) was used to reach absorbance 0.70 ± 0.01 at 734 nm. Samples of 0.01 mL were then diluted with 1 mL of this ABTS radical solution, and absorbance at 734 nm was measured after 6 min at 25 °C [23]. The percentage of ABTS decoloration (%DEC) was calculated as follows: %DEC = 100 × [(Abs_control_ − Abs_sample_)/Abs_control_]. The results were expressed as Trolox Equivalents (mM TE/g, linearity range 0.1–0.8 mM), and as IC_50_.
- Oxygen radical absorbance capacity-pyrogallol red (ORAC-PYR) assay was performed incubating 0.75 mL of 6.6 mM pyrogallol red solution in 75 mM potassium phosphate buffer (pH 7.4) and 0.125 mL of the sample at 25 °C for 10 min. Then 0.125 mL of 0.153 mM 2,2′-azobis(2-amidinopropane) dihydrochloride (APH) solution in 75 mM potassium phosphate buffer (pH 7.4) were added, following the decrease in absorbance at 540 nm for 35 min at 25 °C. The area under the kinetic curve was calculated (AUC_net_) by subtracting the area of the blank (AUC_blank_) from the area of the sample (AUC_sample_): AUC_net_ = AUC_sample_ − AUC_blank_ [23, 24]. The results were expressed as Trolox Equivalents (mM TE, linearity range 0.1–0.8 mM).

### Tyrosinase inhibition

Tyrosinase from *Agaricus bisporus* was purified as already described [27]. Contaminant enzymic activities were carefully checked [28]. Laccase activity was not detectable (<0.001 E.U./mL, using syringaldazine as the substrate [29, 30]). Spectrophotometric assay involving the adduct between 4-tert-butyl-1,2-benzoquinone (TBBQ) and 4-amino-*N*,*N*-diethylaniline (ADA) was performed [31], in order to avoid colorimetric interference by colored extracts (common tyrosinase substrates yield red-yellow products, quite similar to the color of the extracts). The assay mixture contained 50 mM sodium phosphate buffer pH 7.0, 5 mM 4-tert-butylcatechol (TBC), 0.75 mM ADA and 2 E.U. of enzyme in a final volume of 1 mL. The increase in absorbance at 625 nm (ε_625_ = 11,120 M^−1^ cm^−1^ [32]) was followed. 1 Tyrosinase E.U. was defined as the amount of enzyme capable of producing 1 µmol of the adduct between TBBQ and ADA per minute at pH 7 and 25 °C. The amount of inhibition by the test samples was expressed as the concentration necessary to achieve 50 % inhibition (IC_50_).

### Cell line and culture conditions

The B16F10 mouse melanoma cell line, (ICLC ATL 99010) was purchased from the National Institute for Cancer Research c/o CBA (Genoa, Italy). Subcultures of cell line were grown in 75-cm^2^ culture flask in DMEM medium supplemented with 10 % fetal bovine serum (FBS), 2 mM l-Glutamine, 1 % non-essential amino acids, 1 mM Na-pyruvate, penicillin (100 U/mL) and streptomycin(100 µg/mL) at 37 °C in 5 % CO_2_.

### MTT assay for cell viability

The cytotoxic effect of *C. hypocistis* extracts was evaluated in B16F10 melanoma cells using the 3-(4,5-dimethylthiazol-2-yl)-2,5-diphenyltetrazolium bromide (MTT) assay, based on the cleavage of the tetrazolium salt by mitochondrial dehydrogenases in viable cells [33, 34]. MTT is a yellow water-soluble tetrazolium salt. Metabolically active cells are able to convert the dye to water-insoluble dark blue formazan by reductive cleavage of the tetrazolium ring. In brief, 3 × 10^3^ cells/mL in 100 μL of medium were seeded into a 96-well plate and incubated at 37 °C. After 48 h incubation, various concentrations ranging from 25 to 1000 µg/mL of aqueous and ethanolic extracts, were added to cultures and incubated for additional 24 h at 37 °C. An 8 μL portion of MTT solution (5 mg/mL in H_2_O) was then added and left for 4 h at 37 °C. The cells were lysed with 100 μL of DMSO and color development was measured at 570 nm with an auto microplate reader (Infinite 200, Tecan, Austria). The absorbance was proportional to the number of viable cells.

### Statistical analysis

Grafit 7 (Erithacus Software, London UK) and R 2.5.1 software (R Foundation for Statistical Computing, Vienna), were used for statistical analysis. All analyses were performed in triplicate, unless otherwise stated. Data of all experiments are the means and standard deviations of three independent experiments involving triplicate analyses for each sample. Evaluation of the statistical significance of differences was performed using one-way analysis of variation (One-way ANOVA), and Bonferroni post test

## Results and discussion

Despite its numerous applications in traditional medicine [12, 14, 17], the chemical composition of *C.hypocistis* is still almost completely unknown. To give some insight into this, we firstly fractionated the freeze dried plant using three sequential extraction steps with increasingly polar solvents: cyclohexane, ethanol, and water. The hydrophobic portion of the plant was minimal (cyclohexane in fact extracted about 0.5 g per 100 g dried plant), whereas ethanol and water allowed significant recovery of material (28.1 and 16.3 %, respectively).

The full pattern of antimicrobial activities displayed by *C.hypocistis* extract is reported in Table 1. In our hands, cyclohexane extracts were not active. On the contrary, both ethanolic and aqueous extracts displayed an intriguing activity against all the tested Gram-positive bacterial strains. In fact, in all cases inhibition zones were higher than 10 mm when only 0.5 mg of extracts were deposited in each disc. Alves and coworkers [35] suggest that inhibition zones <9 mm correspond to inactive samples, whereas larger inhibition zones correspond to active antimicrobial samples. Particularly, the activity against the clinical isolate methicillin-resistant *S. aureus* is of remarkable interest.

**Table 1:** Antibacterial activity of *Cytinus hypocistis* extracts using disc diffusion method (zone of inhibition in mm)

	Cyclohexane extract 0.5 mg/disc (mm)	Ethanolic extract 0.5 mg/disc (mm)	Water extract 0.5 mg/disc (mm)	Antibiotic control
Gram negative				
*Escherichia coli* DSM 1103	0.0 ± 0.0	0.0 ± 0.0	0.0 ± 0.0	Amp 18.7 ± 0.6
*Enterobacter cloacae* DSM 30054	0.0 ± 0.0	0.0 ± 0.0	0.0 ± 0.0	Oxy 19.7 ± 1.2
*Pseudomonas aeruginosa* DSM 1117	0.0 ± 0.0	0.0 ± 0.0	0.0 ± 0.0	Rif 11.3 ± 0.6
*Acinetobacter baumanii* DSM 30007	0.0 ± 0.0	10.5 ± 3.5	10 ± 2.8	Oxy 15.3 ± 2.3
*Klebsiella pneumoniae* DSM 681	0.0 ± 0.0	0.0 ± 0.0	0.0 ± 0.0	Amp 8.7 ± 0.6
Gram positive				
*Staphylococcus aureus* DSM 1104	0.0 ± 0.0	13.0 ± 0.0	13.5 ± 2.1	Clox 30.5 ± 0.7
*Staphylococcus aureus* MRSA	0.0 ± 0.0	11.5 ± 0.7	11.0 ± 1.4	Clox 0.0 ± 0.0
*Staphylococcus epidermidis* DSM 1798	0.0 ± 0.0	18 ± 4.2	18.0 ± 2.8	Clox 31.5 ± 0.7
*Enterococcus faecalis* DSM 2570	0.0 ± 0.0	10 ± 2.8	9.0 ± 2.8	Amp 23.0 ± 0.0

Rifampicin, cloxacillin, ampicillin, and oxytetracicline, were used as reference antibiotic controls

*Rif* rifampicin (30 µg/disc), *Clox* cloxacillin (5 µg/disc), *Amp* ampicillin (10 µg/disc), *Oxy* oxytetracycline (10 µg/disc)

Compared to the conventional antibiotics cloxacillin and ampicillin, tested under identical conditions, *C.hypocistis* extracts were less active in absolute terms, but displayed a wider spectrum. Significantly, both cloxacillin and ampicillin were inactive against MRSA *S. aureus*. In addition, ethanolic and aqueous extracts were also active against the Gram-negative *A. baumanii* DSM 30007 strain, and the level of potency was comparable to that of oxytetracycline (Table 1). No other data are present in the literature on the antimicrobial properties of *Cytinus*, with the only exception of a report on the activity of methanol extracts on two strains of *Plasmodium falciparum*, with antimalarial activity potentially attributed to hydrolysable tannins [19]. Given the well-known antimicrobial properties of hydrolysable tannins [36], and the composition of *Cytinus* (see below), it is likely that these secondary metabolites might be responsible for the remarkable antibacterial activity of *Cytinus* extracts, as recorded in this study.

To roughly elucidate the composition of the extracts from *C. hypocistis*, the polyphenolic component of the samples was quantified using the Folin-Ciocalteu method for total phenolics, NaNO_2_/AlCl_3_-based assay for the determination of total flavonoids, and the differential pH absorbance method for total anthocyanins. Results are summarized in Table 2. The ethanolic extract was the richest fraction with almost twice the phenolics than the water analogue, whereas the cyclohexane extract was by far the poorest. In all three extracts, flavonoids accounted for only a small part of total phenolics, whereas no anthocyanins were detected. This is in accordance with a previous study of the chemical composition of *Cytinus* [20]. In this paper, *Cytinus* samples collected in Greece were analyzed and hydrolysable tannins (mainly gallic acid derivatives) were identified as the main component [20]. Another hydrolysable tannin of the ellagitannin class, named isoterchebin, was found to be responsible for the yellow pigment of *C. hypocistis* [37].

**Table 2:** Total antioxidant capacity of extracts from *Cytinus hypocistis*

Assay	Cyclohexane extract	Ethanolic extract	Water extract
ORAC-PYR (mTE/g)	1.5 ± 0.4	28.1 ± 2.5	13.7 ± 1.3
DPPH (mTE/g)	0.20 ± 0.03	2.25 ± 0.07	1.01 ± 0.07
DPPH (IC_50_ µg/mL)	95.0 ± 0.2	24.2 ± 0.5	24.5 ± 0.8
TEAC (mTE/g)	0.64 ± 0.09	7.22 ± 0.08	5.32 ± 0.02
TEAC (IC_50_ mg/mL)	0.800 ± 0.132	0.071 ± 0.009	0.089 ± 0.002
FRAP (mTE/g)	0.27 ± 0.01	6.70 ± 0.03	6.69 ± 0.09
FRAP (mmol Fe^II^/g)	1.20 ± 0.01	22.5 ± 0.1	20.9 ± 0.1
Total phenolics (mGAE/g)	1.22 ± 0.12	7.82 ± 0.01	4.32 ± 0.05
Total flavonoids (mCE/g)	0.18 ± 0.01	0.63 ± 0.01	0.69 ± 0.02
Total anthocyanin (mg cyanidin 3-*O*-glucoside/g)	n.d.	n.d.	n.d.

Data are the means of at least three independent determinations ± SD

*n.d.* not detectable

*Cytinus hypocistis* extracts were then evaluated for their antioxidant activity using three different electron-transfer-based (ET) methods. Since the ET methods are able to detect only reducing capacity, and not complete antioxidant activity [24, 26], a spectrophotometric HAT method (ORAC-PYR) was also included in the study. In accordance with this observation, the results displayed in Table 2 show that ORAC-PYR gave in all cases the highest antioxidant capacity for all the tested samples, as already demonstrated for other plant samples [23, 33]. In strict agreement with the phenolics composition, the cyclohexane extract showed the poorest antioxidant activity, whereas the alcoholic extract presented about a twofold higher ORAC-PYR activity than the water sample. To the best of our knowledge, this is the first report about polyphenolic composition and antioxidant activity for *C. hypocistis*, and no comparison is possible among the same genus or even family. As reported above, the few available studies describe hydrolysable tannins as important constituents of *Cytinus* samples [20, 37]. We rather suggest the comparison with *Cynomorium coccineum* (Cynomoriaceae, Fig. 1c, d), another parasitic and non-photosynthetic plant occurring in the same geographic area, which has been already fully analyzed about chemical composition and antioxidant capacity [23, 33, 38]. *C. coccineum* antioxidant capacity of both water and alcoholic extracts showed quite similar values for both samples (ORAC-PYR was respectively 1.18 and 0.91 mM TE/g) [23]. The *C. hypocistis* alcoholic extract was on the contrary significantly more active than the water extract, suggesting a different chemical composition compared to *C. coccineum*, where gallic acid and cyanidin 3-*O*-glucoside were the main constituents [23]. It should be noted that in *C. hypocistis* extracts no cyanidins or anthocyanins were detected using the described spectrophotometric assay [25].

Besides, *C. hypocistis* extracts showed a greater antioxidant activity than *C. coccineum* samples; antioxidant activity of ethanolic extract was almost 20-fold higher, taking into account ORAC-PYR assay [23]. However, in the cited paper, a different extraction procedure was performed. When the same protocol used in the current study was applied, *C. coccineum* water extract gave 6.8 mM TE/g ORAC-PYR activity (Zucca et al., in preparation), being in any case significantly lower than both *C. hypocistis* alcoholic and water extracts. According to the obtained data, *C. hypocistis* can be assessed as a valuable source of antioxidant chemicals, even for food formulations.

Tyrosinase (or polyphenoloxidase PPO) is a well-known enzyme involved in melanogenesis and food browning. Accordingly, the development of new inhibitors of this enzymic activity is a very productive field of research [39–41]. To avoid any interference with the tyrosinase inhibition tests, both mono- and di-phenolase activity was ruled out in the *C. hypocistis* extracts, using common activity assays already reported [27, 31]. All the extracts were able to inhibit PPO activity, albeit at very different extents. Particularly, the cyclohexane extract was about one order of magnitude less effective (IC_50_ 263 μg) than water and ethanolic extracts. The latter presented the lowest IC_50_ (4.01 μg), being even lower than *C. coccineum* water extract (15.6 μg) (Zucca et al., in preparation). These data are quite promising as well in the perspective of *C. hypocistis* application in food formulations as an anti-browning agent.

**Fig. 2:**
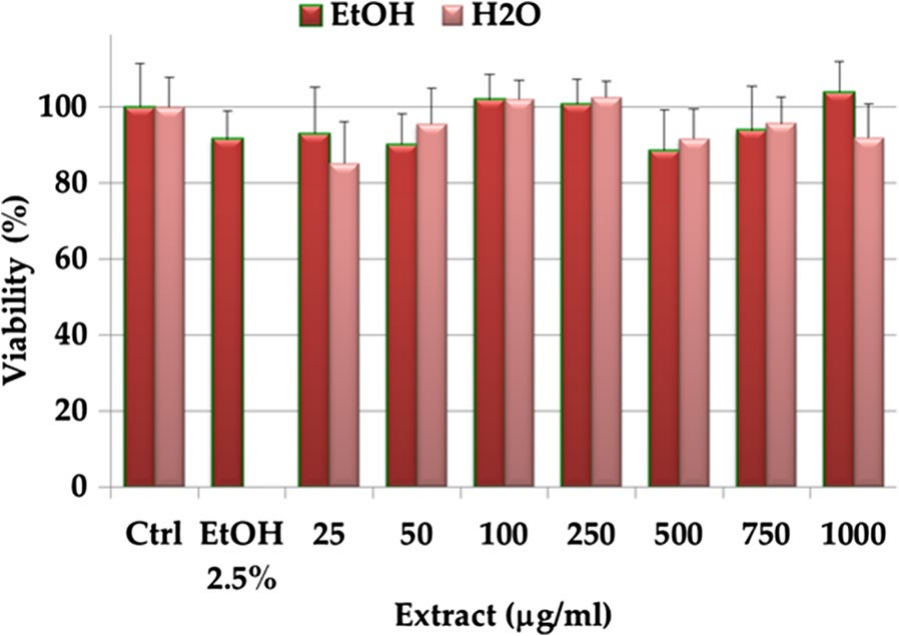
Viability (expressed as % of the control) (MTT assay) induced by 24 h incubation with *C. hypocistis* aqueous and ethanolic extracts (25–1000 μg/mL) in melanoma B16F10 cells. Data are the means ± standard deviations of three independent experiments involving triplicate analyses for each sample

*Cytinus hypocistis* extracts have been also evaluated for their ability to affect the viability of tumoral cell lines. In accordance, the relationship between concentration of extracts and cell viability of B16F10 melanoma cells was investigated by the MTT assay. B16-F10 cells were treated with aqueous and ethanolic extracts of *C. hypocistis* at concentrations ranging from 25 to 1000 μg/mL for 24 h at 37 °C. We have chosen such a range of concentrations on the basis of similar experiments performed with water extracts and fixed oil from *C. coccineum* [33, 38]. In *C. coccineum* the same concentrations showed significant toxicity against B16F10 cells. The results of the MTT cell viability assay showed that treatment with both the extracts, in this case, did not have a significant cytotoxic effect even at the highest dose (1000 µg/mL) on this tumoral cell line (Fig. 2). Our data are at variance with that reported by Magiatis and coworkers [20], who found that the methanolic extracts Greek *Cytinus* exhibited cytotoxic activity against several cancer cell lines, although B16F10 melanoma cells were not tested. The authors attributed this cytotoxic activity to hydrolysable tannins, without any obvious dependence on their molecular weights [20]. Future experiments will be needed to investigate the effect on different (not tumor) cell lines and to validate these results for possible pharmaceutical (i.e. antimicrobial), nutraceutical, and/or cosmetic use.

## Conclusions

On the whole, *Cytinus* is a plant endowed with intriguing antibacterial, antioxidant and anti-tyrosinase properties, as encompassed in Table 3. The reported data are promising in the perspective of the confirmation of the ethnobotanical application of the plants. Further efforts will be however devoted in the near future in order to isolate and fully characterize biologically active compound(s).

**Table 3:** Summary of the biological activities detected in this paper for *C. hypocistis* extracts

	Cyclohexane extract	Ethanolic extract	Water extract
Amount (g/100 g of dried plant material)	0.5 %	28.1 %	16.3 %
Antioxidant activity	±	+++	++
Antibacterial activity (Gram-positive)	–	++	++
Antibacterial activity (Gram-negative)	–	±	±
Anti-tyrosinase activity	±	+++	++
Cytotoxicity against B16F10 melanoma cells	–	–	–

## Abbreviations

DPPH: 1,1-diphenyl-2-picrylhydrazyl
TEAC: trolox equivalent antioxidant capacity
FRAP: ferric reducing antioxidant power
APH: 2,2′-Azo-bis(2-amidopropane)dihydrochloride
ORAC: oxygen radical absorbance capacity
GAE: gallic acid equivalents
TE: trolox equivalents
MTT: 3-(4,5-dimethylthiazol-2-yl)-2,5-diphenyltetrazolium bromide
